# Essential Fatty Acids and Their Metabolites in the Pathobiology of Inflammation and Its Resolution

**DOI:** 10.3390/biom11121873

**Published:** 2021-12-14

**Authors:** Undurti N Das

**Affiliations:** 1 UND Life Sciences, 2221 NW 5th St., Battle Ground, WA 98604, USA; undurti@hotmail.com or undurti@lipidworld.com; Tel.: +1-508-904-5376; 2 International Research Centre, Biotechnologies of the Third Millennium, ITMO University, Saint Petersburg 191002, Russia

**Keywords:** SARS-CoV-2, COVID-19, polyunsaturated fatty acids, arachidonic acid, prostaglandins, lipoxin A4, resolvins, protectins, maresins, inflammation, ARDS

## Abstract

Arachidonic acid (AA) metabolism is critical in the initiation and resolution of inflammation. Prostaglandin E2 (PGE2) and leukotriene B4/D4/E4 (LTB4/LD4/LTE4), derived from AA, are involved in the initiation of inflammation and regulation of immune response, hematopoiesis, and M1 (pro-inflammatory) macrophage facilitation. Paradoxically, PGE2 suppresses interleukin-6 (IL-6) and tumor necrosis factor-α (TNF-α) production and triggers the production of lipoxin A4 (LXA4) from AA to initiate inflammation resolution process and augment regeneration of tissues. LXA4 suppresses PGE2 and LTs’ synthesis and action and facilitates M2 macrophage generation to resolve inflammation. AA inactivates enveloped viruses including SARS-CoV-2. Macrophages, NK cells, T cells, and other immunocytes release AA and other bioactive lipids to produce their anti-microbial actions. AA, PGE2, and LXA4 have cytoprotective actions, regulate nitric oxide generation, and are critical to maintain cell shape and control cell motility and phagocytosis, and inflammation, immunity, and anti-microbial actions. Hence, it is proposed that AA plays a crucial role in the pathobiology of ischemia/reperfusion injury, sepsis, COVID-19, and other critical illnesses, implying that its (AA) administration may be of significant benefit in the prevention and amelioration of these diseases.

## 1. Introduction

Inflammation and its timely resolution are essential for health and disease, especially to recover from microbial infections, injury, and surgery. Some of the serious illnesses in which inflammation plays a critical role include ischemia/reperfusion injury, sepsis, ARDS (acute respiratory distress syndrome), post-surgical sepsis/shock, and cytokine storm seen in patients with COVID-19. In all these conditions, acute inflammation goes unabated due to excess production of pro-inflammatory cytokines, prostaglandins, leukotrienes, bradykinin, and reactive oxygen species (ROS). In addition, there could occur concomitant deficiency of anti-inflammatory cytokines and lipid mediators. Severe COVID-19 (coronavirus disease 2019) caused by SARS-CoV-2, can cause ARDS because of damage to endothelial cells of various blood vessels, especially pulmonary vessels resulting in endotheliopathy, alveolar exudative inflammation and interstitial inflammation, alveolar epithelium proliferation, and hyaline membrane formation. One of the best examples of critical illnesses is COVID-19, which is associated with the excess production of the pro-inflammatory cytokines interleukin-6 (IL-6), tumor necrosis factor-α (TNF-α,) and other cytokines. COVID-19 has been shown to affect almost all vital organs in the body from head to toe including respiratory, cardiovascular, gut, renal, central nervous, immune, and coagulation systems. Respiratory failure that may occur in those with severe COVID-19 calls for invasive respiratory support and antithrombotic measures. In this review, COVID-19 is discussed in detail as an example of the severe critical illnesses and inflammatory and immune changes that occur in it and other illnesses such as ischemia/reperfusion injury, sepsis, and ARDS and the potential role of bioactive lipids and their pro- and anti-inflammatory metabolites. Based on this evidence, it is suggested that AA and its metabolites may have a therapeutic role in the prevention and management of ischemia/reperfusion injury, sepsis, COVID-19, ARDS, and other critical illnesses, with emphasis on COVID-19.

## 2. SARS-CoV-2 and COVID-19

The SARS-CoV-2 virus binds to the angiotensin-converting enzyme 2 (ACE2) receptor expressed by nasal and bronchial epithelial cells and pneumocytes by the ability of its spike (S) protein to bind to the same (ACE2). The type 2 transmembrane serine protease (TMPRSS2) of the host cell can cleave ACE2 and activate the S protein and promote SARS-CoV-2 virus uptake into the host cells [1]. Both ACE2 and TMPRSS2 need to be expressed by the host target cells including the alveolar epithelial type II and vascular endothelial cells so that SARS-CoV-2 can infect and induce inflammatory reaction, leading to the influx of inflammatory cells such as monocytes and neutrophils that, in turn, release pro-inflammatory cytokines (IL-1, IL-6, TNF-α, HMGB1, etc.) and reactive oxygen species [2]. These events lead to ground-glass opacities on computed tomography (CT) and pulmonary edema of alveolar spaces with hyaline membrane formation that leads to ARDS. In addition to pro-inflammatory cytokines, a potential role of bradykinin has been proposed in these features [3]. Thus, COVID-19 is characterized by endothelial barrier disruption, alveolar-capillary oxygen transmission dysfunction, impaired oxygen capacity diffusion, diffuse intravascular coagulation and pulmonary microthrombi, and a high incidence of thrombotic complications including deep venous thrombosis, pulmonary embolism, and arterial thrombosis that can result in limb ischemia, ischemic stroke, and myocardial infarction.

COVID-19 is like other respiratory viral diseases such as influenza and infects and induces apoptosis of T lymphocytes to induce severe lymphopenia and impair lymphopoiesis partly due to a striking reduction in Bcl-6+ germinal center B cells that correlate with an early specific block in Bcl-6+ TFH cell differentiation associated with an increase in T-bet+ TH1 cells and aberrant extrafollicular TNF-α accumulation in the spleen and lymph nodes [4]. These results suggest that the high TNF-α levels seen in severe COVID-19 not only cause a so-called ‘cytokine storm’, which is now rather controversial [5], but also suppress immune response, leading to a lack of type I IFNs, reduced HLA-DR in the myeloid cells, and transient expression of IFN-stimulated genes [6,7]. Since developing specific anti-SARS-CoV-2 compounds and vaccine(s) is time consuming, especially when the SARS-CoV-2 is mutating, making it resistant/unresponsive/less responsive to the vaccines that have been developed, it is important to develop alternative strategies to inactivate the virus and prevent or ameliorate inflammatory events that are associated with SARS-CoV-2 infection. I propose that arachidonic acid (AA, 20:4 n-6), a precursor of pro-inflammatory prostaglandin E2 (PGE2) and anti-inflammatory lipoxin A4 (LXA4), and other bioactive lipids have a significant role not only in the pathobiology of COVID-19 but also in sepsis, ischemia-reperfusion injury, autoimmune arthritis (and possibly, in lupus), and in their prevention and management. 

## 3. EFAs’ (Essential Fatty Acids’) Metabolism 

**Figure 1:**
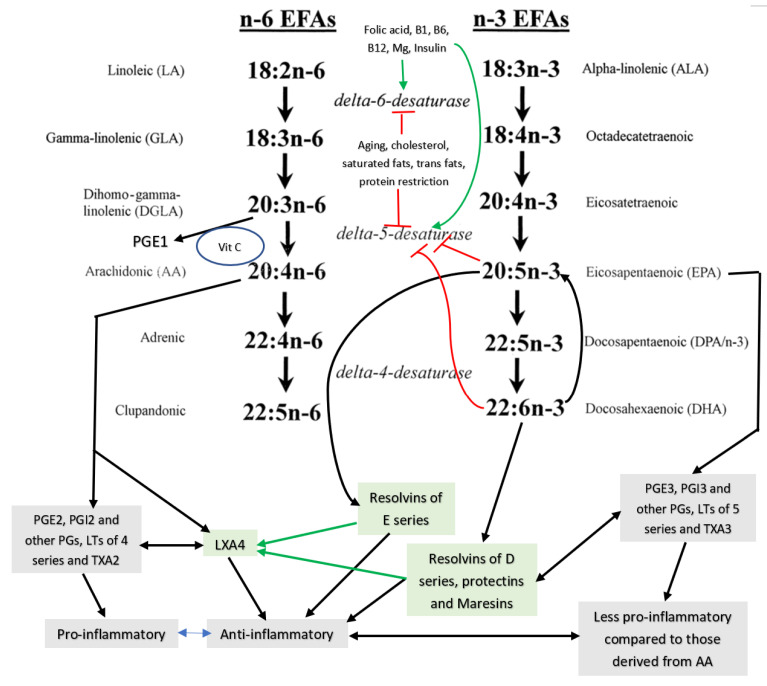
Metabolism of essential fatty acids (EFAs).

Dietary EFAs’ *cis*-linoleic acid (LA, 18:2 n-6) and α-linolenic acid (ALA, 18:3 n-3) are widely distributed in our diet and, hence, their deficiency is rare in humans. LA is metabolized by delta-6- desaturase and delta-5- desaturase and elongases to convert it to γ-linoleic acid (GLA, 18:3 n-6), dihomo-GLA (DGLA, 20:3 n-6), and arachidonic acid (AA, 20:4 n-6), whereas ALA gives rise to eicosapentaenoic acid EPA, 20:5 n-3, and docosahexaenoic acid (DHLA, 22:6 n-3) (see Figure 1 for metabolism of EFAs). Despite the proposal that both LA and ALA are metabolized by the same set of desaturases (delta-6-desaturase and delta-5-desaturase), there is indirect evidence that isoforms of desaturases may exist that may metabolize LA and ALA differently. This may explain why, under some specific conditions, GLA, DGLA, and AA levels are decreased but EPA and DHA may not show a similar degree of decrease or may, in fact, be increased. Hence, particular attention needs to be applied in interpreting the plasma and/or tissue levels of changes in EFAs and their metabolites in several diseases. For instance, it was reported that in Yoshida hepatoma cells, a drastic reduction in the concentrations of AA was noted with little or no change in DHA (see Table 1). If the same desaturases were to be acting on both LA and ALA to form their long-chain metabolites, one would expect the same degree of decrease in the concentrations of AA and DHA. However, this was not the case [8]. Similarly, the concentrations of n-6 fatty acids, especially GLA, DGLA, and AA, were found to be decreased to a greater degree compared to n-3 fatty acids such as EPA and DHA in those with type 2 diabetes mellitus (T2DM), hypertension (HTN), diabetic nephropathy (DN), and coronary heart disease (CHD) (see Table 2) [9]. This once again supports the contention that there could occur isoenzymes of desaturases. This suggestion applies to the changes observed in the plasma and tissue levels of their products such as prostaglandins (PGs), leukotrienes (LTs), thromboxanes (TXs), lipoxins (LXs), resolvins (RSVs), protectins (PRTs), and maresins (MaRs). These desaturases are the rate-limiting steps in the metabolism of LA and ALA. It is noteworthy that several co-factors are needed for adequate activity of desaturases. Some of these factors include magnesium; insulin; vitamins C, B1, B6, and B12; and folic acid, whereas high glucose inhibits their activity. DGLA is the precursor of 1 series prostaglandins (PGs) such as PGE1, whereas AA is the precursor of 2 series of PGs, thromboxanes (TXs), and 4 series leukotrienes (LTs), while 3 series PGs, TXs, and 5 series LTs are derived from EPA. Most of these PGs, TXs, and LTs are pro-inflammatory in nature. It is noteworthy that 2 series PGs, TXs, and 4 series LTs are more pro-inflammatory in nature compared to 3 series PGs, TXs, and 5 series LTs. Thus, PGE2 is more potent compared to PGE3 in inducing inflammatory events [10,11,12]. PGE1 derived from DGLA has significant anti-inflammatory actions [10].

## 4. Interaction among Various EFAs and Their Pro- and Anti-Inflammatory Metabolites 

**Figure 2:**
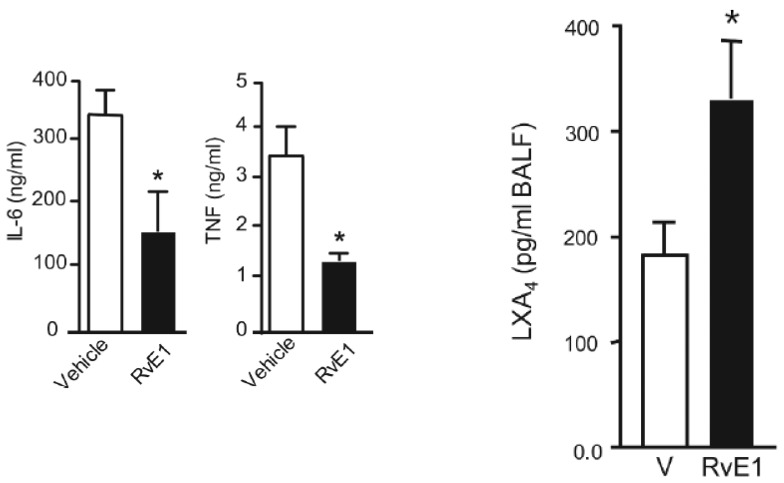
Effect of resolvin E1 on IL-6 and TNF-α levels and LXA4 formation in bronchial alveolar fluid in animals with allergic airway inflammation (taken from Ref. [16]). * *p* < 0.05 compared to vehicle (V). In this study, the animals received 50 or 100 ng/day intravenously.

**Figure 3:**
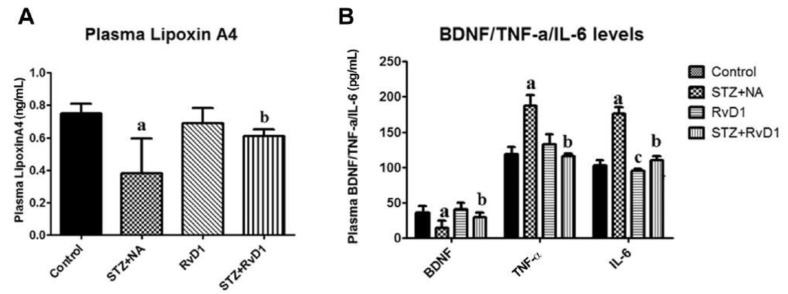
Effect of resolvin D1 (60 ng/animal by intraperitoneal route) on STZ-induced type 2 diabetes mellitus. Resolvin D1 not only abrogated STZ-induced diabetes but also suppressed STZ-induced increase in plasma IL-6 and TNF-α levels and restored plasma LXA4 and BDNF to normal (these data are taken from ref. [14]). (**A**) ^a^ *p* ≤ 0.001 compared to untreated control; ^b^ *p* ≤ 0.01 compared to streptozotocin + nicotinamide (STZ + NA) group. (**B**) ^a^ *p* ≤ 0.01 compared to STZ + NA, and ^b^ *p* ≤ 0.01 compared to untreated control. TNF-α studies, ^a^ *p* ≤ 0.01 compared to control, and ^b^ *p* ≤ 0.05 compared to STZ + NA. IL-6 studies; ^a^ *p* ≤ 0.01 and ^c^ *p* ≤ 0.01 compared to control. ^b^ *p* ≤ 0.01 compared to STZ + NA group. All values are expressed as mean ± SEM.

**Figure 4:**
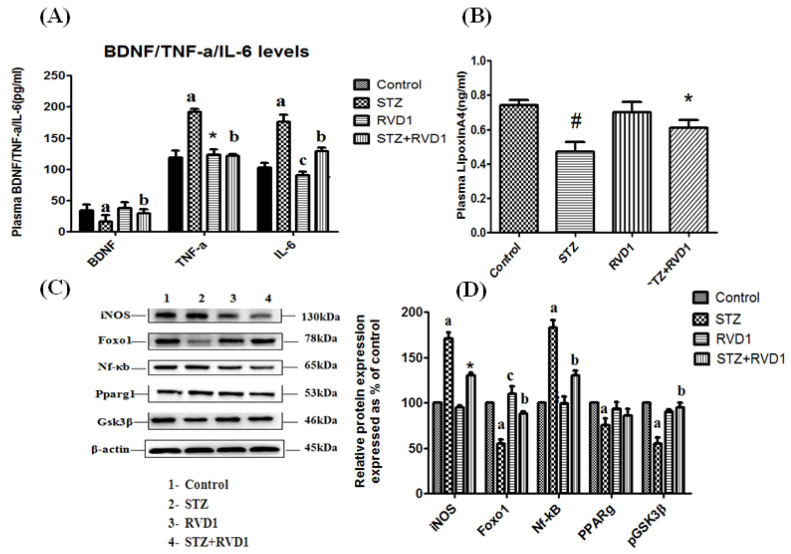
Effect of RVD1 treatment on plasma levels of BDNF/TNF-α/IL-6/LXA4 and protein expression in pancreatic tissue samples. (**A**) Plasma BDNF/TNF-α/IL-6 levels in STZ + RVD1- vs. STZ (T1DM)-treated groups estimated at the end of the study (day 30). ^b^ *p* ≤ 0.01 compared to STZ (T1DM), and ^a^ p ≤ 0.01 compared to untreated control; TNF-α studies: ^a^ *p* ≤ 0.001; * *p* ≤ 0.01 compared to control and compared to STZ control; and *^b^ p* ≤ 0.05 compared to STZ (T1DM); IL-6 studies: ^a^ *p* ≤ 0.01 and ^c^ p ≤ 0.05 compared to untreated control and STZ control values. ^b^ *p* ≤ 0.01 compared to STZ (T1DM) group. All values are expressed as mean ± SEM. (**B**) Measurement of LXA4 levels in the plasma of various groups measured at the end of the study (day 30). # *p* ≤ 0.001 compared to untreated control. * *p* ≤ 0.01 compared to STZ (T1DM) control (positive control group). (**C**,**D**) Protein expression studies in pancreatic tissue of the rats of various groups. Total protein extracted from the pancreatic tissue samples were collected at the end of the study (day 30) and used for Western blots for Nf-κb, Foxo1, PPAR-γ, *p*-GSK3β, iNOS, and beta Actin. Equality of loading of the samples was confirmed by beta actin protein expression. All values are expressed as mean ± SEM. ^a^ *p* ≤ 0.01 and ^c^ *p* ≤ 0.05 compared to control values. *^b^ p* ≤ 0.01 compared to STZ (T1DM) (these data are taken from Reference [15]). It is evident from this data that resolvin D1 not only prevents STZ-induced type 1 DM in experimental animals but also suppresses inflammation, as evidenced by decreased plasma IL-6 and TNF-α levels and decreased expression of NF-kB and iNOS, and an increase in the circulating levels of LXA4 that was suppressed by STZ treatment. Thus, at least, in part, the anti-inflammatory actions of resolvin D1 are mediated by increased formation of LXA4.

**Figure 5:**
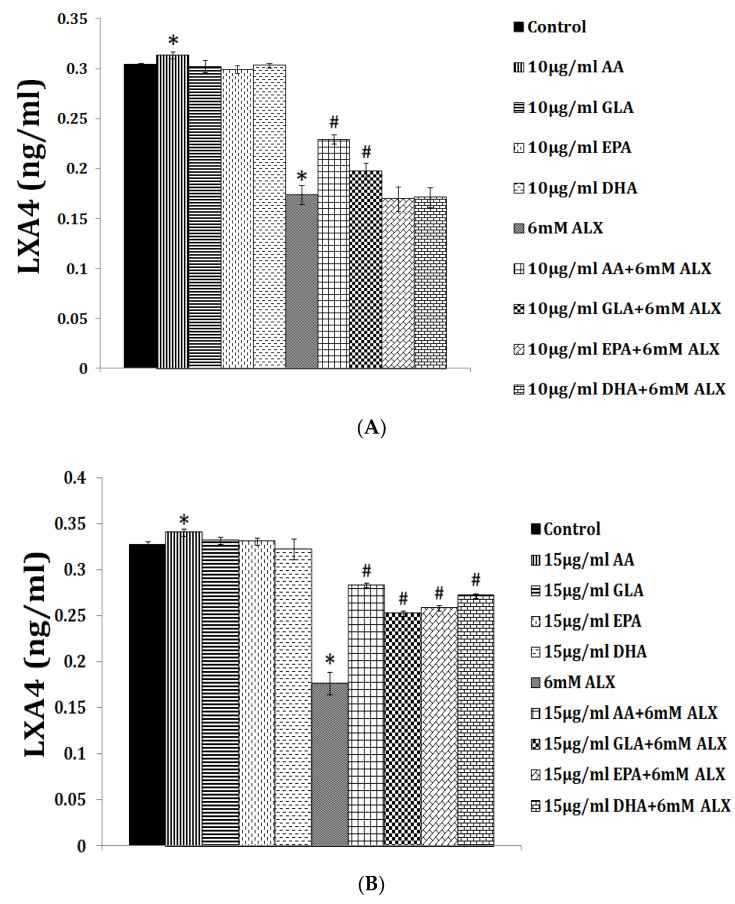
Effect of alloxan on LXA4 secretion by RIN cells and its modulation by various PUFAs in vitro. ALX = Alloxan. (**A**) RIN5F (rat insulinoma) cells were treated with 10 µg/mL PUFAs ± ALX (6 mM) for 1 h. The LXA4 was estimated in the supernatant of the cell cultures. All the above sets of experiments were done in triplicate on two separate occasions (*n* = 6) and values are expressed as mean ± SEM. * *p* ≤ 0.05 compared to untreated control, # *p* ≤ 0.05 compared to ALX. (**B**) RIN5F cells were treated with 15 µg/mL of PUFAs ± ALX (6 mM) for 1 h. The LXA4 was estimated in the supernatants of the cell cultures. All the above sets of experiments were done in triplicate on two separate occasions (*n* = 6) and values are expressed as mean ± SEM. * *p* ≤ 0.05 compared to untreated control, # *p* ≤ 0.05 compared to alloxan (ALX). It is seen that at a 10-µg/mL dose of EPA and DHA treatment there was no increase in LXA4 secretion by RIN5F cells in vitro in the presence of ALX (6 mM) (**A**). However, when RIN5F cells were supplemented with 15 µg/mL of EPA and DHA, there was a significant increase LXA4 secretion, even in the presence of ALX (6 mM). These data are taken from [13].

**Figure 6:**
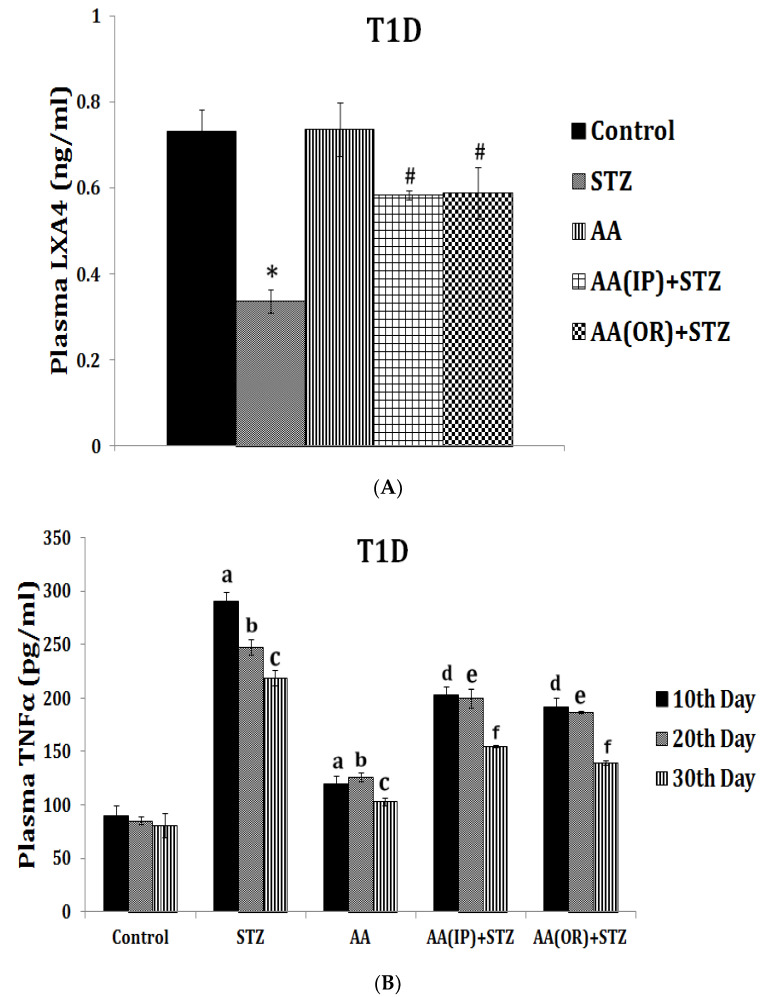
Effect of arachidonic acid (AA), the precursor of LXA4 and PGE2, on STZ-induced type 1 diabetes mellitus in Wistar rats. Both oral and intraperitoneal (i.p.) administrations of AA (10 μg/animal for 7 days) not only prevented STZ-induced type 1 diabetes mellitus and restored plasma glucose and insulin to normal but also enhanced plasma LXA4 levels to near control values (**A**) and suppressed TNF-α levels (**B**). These results suggest that AA is as potent as LXA4 in preventing diabetes and suppressing inflammation at least, in part, by enhancing LXA4 formation. Thus, AA is a potent anti-inflammatory molecule similar to LXA4. These data are taken from ref. [17]. Similar results were obtained with AA and LXA4 in a STZ-induced type 2 diabetes mellitus animal model (see reference [13]). (**A**) Measurement of LXA4 levels in plasma of AA ± STZ-treated animals at the end of the study (day 30). (**B**) Plasma TNF-α level in AA ± STZ treated rats. TNF-α measurement was done in plasma collected once every 10 days until the end of the study. All values are expressed as mean ± SEM. ^a^ *p* ≤ 0.05 compared to the 10th day of control. ^b^ *p* ≤ 0.05 compared to the 20th day of control. ^c^ *p* ≤ 0.05 compared to the 30th day of control. ^d^ *p* ≤ 0.05 compared to the 10th day of STZ control. ^e^ *p* ≤ 0.05 compared to the 20th day of STZ control. ^f^ *p* ≤ 0.05 compared to the 30th day of STZ control. * *p* ≤ 0.05 compared to untreated control. # *p* ≤ 0.05 compared to STZ control. All the above studies were done wherein each group consisted of *n* = 6 and all values are expressed as mean ± SEM.

**Figure 7:**
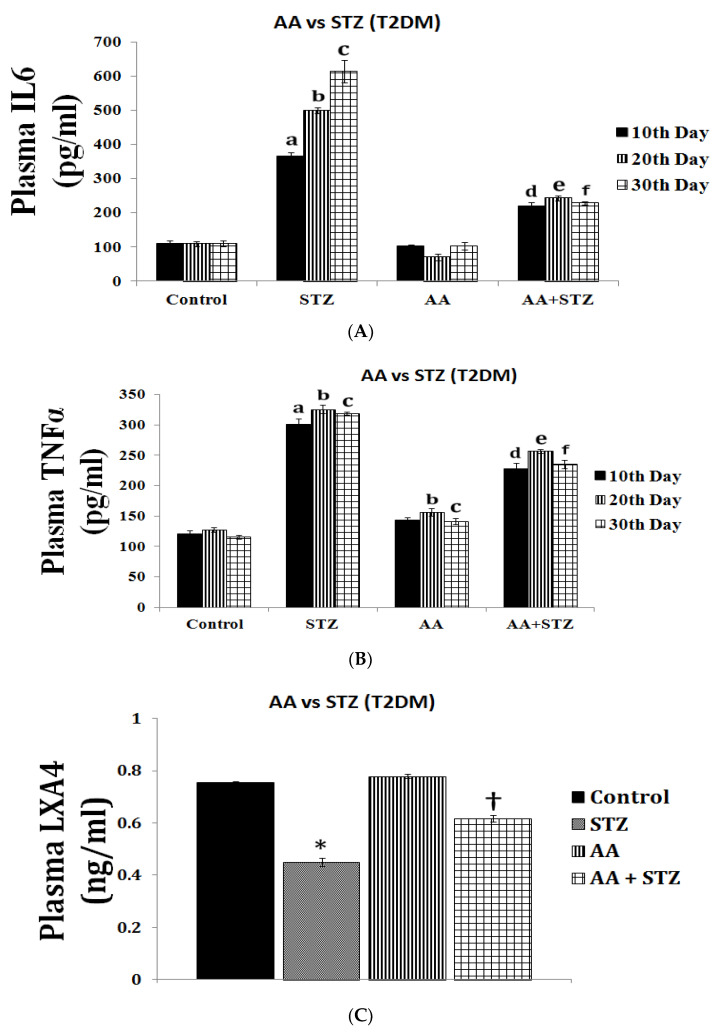

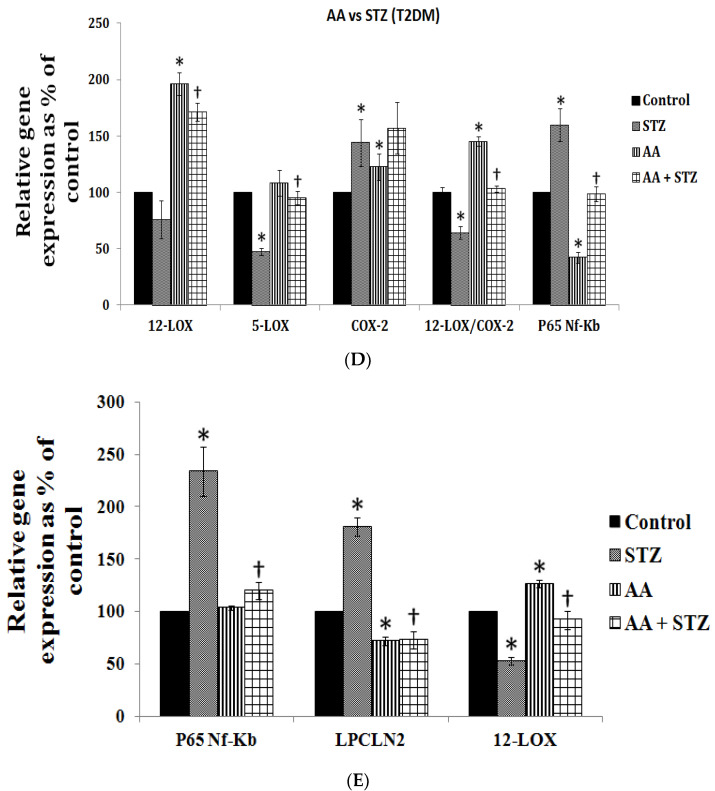
Effect of AA (orally 10 μg/animal for 1 week) on STZ-induced type 2 diabetes mellitus, plasma IL-6 and TNF levels, and expression of pro-inflammatory genes in Wistar rats. AA not only prevented the development of diabetes mellitus but also suppressed plasma IL-6 and TNF-α levels (**A**,**B**) and showed enhanced plasma LXA4 levels (**C**) and the expression of pro-inflammatory NF-κB (in the pancreas) (**D**) and LPCLN (lipocalin-2) genes in the adipose tissue (**E**) and restoration to normal the expression of 5-LOX (in the pancreas) (**D**) and NF-κB (adipose tissue, (**E**) genes. Increase in the expression of COX-2 and 12-LOX genes is not surprising since these are needed for the formation of LXA4 from AA. These data are taken from ref. [17]. A. Plasma IL-6 was measured in all animals using a Rat IL-6 ELISA Kit by Abcam (ab100772) Cambridge, USA. IL-6 measurement was done in plasma collected once every 10 days until the end of the study. B. Plasma TNF-α was measured in all animals. Quantikine TNF-α Immunoassay ELISA kit (R & D Systems, Minneapolis, MN, USA). TNF-α measurement was done in plasma collected once every 10 days until the end of the study. All Values in (**A**,**B**) are expressed as mean ± SEM. ^a^ *p* ≤ 0.05 compared to the 10^th^ day of control; ^b^ *p* ≤ 0.05 compared to the 20th day of control; ^c^ *p* ≤ 0.05 compared to the 30th day of control; ^d^ *p* ≤ 0.05 compared to the 10th day of STZ control; ^e^ *p* ≤ 0.05 compared to the 20th day of STZ control; ^f^ *p* ≤ 0.05 compared to the 30th day of STZ control. C. Measurement of LXA4 levels in plasma of all the groups of animals was performed at the end of the study (day 30). All the values are expressed as mean ± SEM. * *p* ≤ 0.05 compared to untreated control, † *p* ≤ 0.05 compared to STZ. (**D**,**E**). Gene expression studies in pancreas and adipose tissues. Gene expression studies were performed in the pancreas (**D**) and adipose (**E**) tissues collected at end of the experiment. In pancreas, the percentage of change in the expression of genes: 5-LOX, 12-LOX, COX-2, and Nf-kB were studied using the semi-quantitative polymerized chain reaction (PCR) method. The equality of sample loading was confirmed by beta actin gene expression. Quantification of genes was done by Major Science image analysis software. In adipose tissue, the percentage of change in the expression of genes 12-LOX, LPCLN2, and Nf-kB was studied using the semi-quantitative polymerized chain reaction (PCR) method. The equality of sample loading was confirmed by beta actin gene expression. Quantification of genes was done by Major Science image analysis software. All the values are expressed as mean ± SEM. * *p* ≤ 0.05 compared to untreated control, † *p* ≤ 0.05 compared to STZ.

LXA4 (lipoxin A4), a potent anti-inflammatory molecule, is derived from AA. Similar anti-inflammatory molecules, E series resolvins, are derived from EPA, whereas resolvins of D series, protectins, and maresins are formed from DHA [10]. The fact that both pro- and anti-inflammatory molecules are derived from the same precursors (especially AA and EPA) indicates that there could occur a positive and negative feedback regulation among them, a balance that may play a critical role in the regulation of inflammation in various conditions. It is noteworthy that pro-inflammatory PGs of 2 series and LTs of 4 series derived from AA are more potent compared to PGs of 3 series and LTs of 5 series derived from EPA. By the same token, it is interesting that LXA4 appears to be more potent than resolvins, protectins, and maresins derived from EPA and DHA [8,10]. In addition, there is a crosstalk among various PGs, LTs, LXA4, resolvins, protectins, and maresins. For instance, LXA4 inhibits the production of PGE2 and LTs, a mechanism by which it behaves as an anti-inflammatory compound. PGE1, PGE2, LXA4, resolvins, protectins, and maresins inhibit the production of pro-inflammatory cytokines especially interlukin-6 (IL-6) and tumor necrosis factor-α (TNF-α). Our studies revealed that, in all probability, LXA4 is more potent in suppressing the production of IL-6 and TNF-α compared to resolvins and protectins, especially in streptozotocin-induced type1 and type 2 diabetic models [13,14,15]. In these studies [13,14,15], it was noted that when the same concentrations of LXA4 and resolvins were employed, the amount of decrease in plasma IL-6 and TNF-α concentrations was more impressive in LXA4-treated animals compared to those treated with resolvins, implying that the former (LXA4) is more potent in bringing about its anti-inflammatory action compared to the latter (LXA4 > resolvins). Hence, it is reasonable to suggest that LXA4 is more potent than resolvins, protectins, and maresins in suppressing inflammation. It is interesting to note that both resolvin D1 and E1, derived from DHA and EPA, respectively, enhance the production of LXA4 (see Figure 2, Figure 3 and Figure 4) (see references [14,15,16]), implying that, to some extent, resolvins (and probably protectins and maresins) bring about their anti-inflammatory actions by enhancing the formation of LXA4 that is derived from AA. It is noteworthy that administration of AA enhanced LXA4 formation both in vitro and in vivo (see Figure 5, Figure 6 and Figure 7) and suppressed the expression of pro-inflammatory genes such as NF-kB and lipocalin [13,17]. It is of considerable interest to note that other fatty acids such as GLA, EPA, and DHA enhanced the production of LXA4 from pancreatic beta cells in vitro that was suppressed by alloxan treatment (see Figure 5). Although the exact mechanism by which these fatty acids enhance LXA4 formation is not clear, it may, partly, be by their ability to displace AA from the cell membrane lipid pool. These results attest to the proposal that there is a close interaction between n-6 and n-3 fatty acids and their metabolism (see Figure 1). 

This concept is further supported by the known inverse relationship between EFAs and their metabolites in adipose tissue, especially LA, DGLA, AA, and EPA, and mortality due to CHD [18,19,20,21,22,23]. These results imply that increased consumption of LA and EPA can prevent CHD that could be attributed to increased formation of PGE1 from DGLA (derived from LA) and PGI3 (derived from EPA), both of which are vasodilators and platelet anti-aggregators [24,25]. Studies showed that infusion of DGLA strongly stimulates the release of PGI3 from EPA, a potent vasodilator and platelet anti-aggregator, possibly, via the formation of a hydroperoxide derivative of DGLA [25]. These results suggest that the presence of adequate amounts of both n-6 and n-3 fatty acids is needed to derive their beneficial actions.

## 5. EFAs and Their Metabolites in Inflammation 

**Figure 8:**
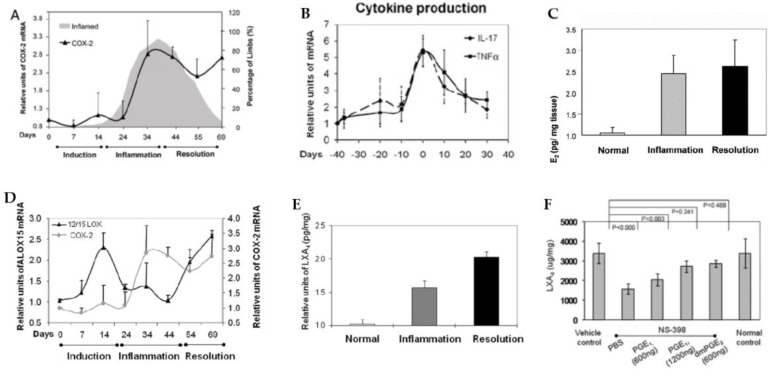
Changes in the expression of COX-2 and cytokines (IL-17 and TNF-α) in a murine model of autoimmune arthritis. (**A**,**B**) PGE2 levels, (**C**) expression of LOX-15, (**D**) changes in the concentration of LXA4, and (**E**) effect of various concentrations of PGE2 on LXA4 production (**F**) in the joints of murine autoimmune arthritis model (see reference [36]). These data were taken from reference [36] and modified. It is evident from this data that, during the acute inflammation stage, there is enhanced expression of COX-2, increased production of IL-17 and TNF-α, augmentation in the production of PGE2, and relatively less production of LXA4 and 12-15-LOX expression. During the phase of resolution of inflammation, the expression of COX-2 (**A**) and PGE2 (**C**) production remains high, whereas 12-15-LOX expression is decreased (**D**) and LXA4 production is elevated (**E**) with concomitant decrease in IL-17 and TNF-α production (**B**) that results in resolution of joint inflammation. Both PGE1 and PGE2 enhanced the production of LXA4 (PGE2 > PGE1) (**F**) by enhancing the expression of ALX, the receptor of LXA4.

**Figure 9:**
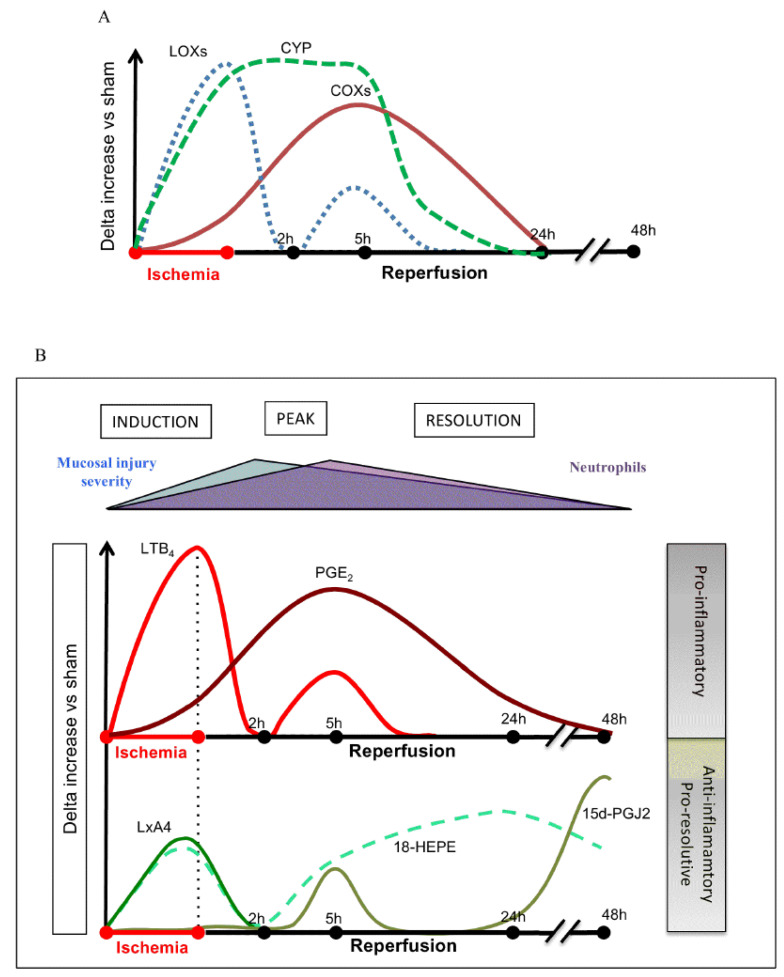
Changes in the concentrations of various bioactive lipids (specifically products of AA, EPA, and DHA) in ischemia reperfusion injury in mouse intestine. These results are somewhat like those seen in Figure 7 except for the fact that, in this study, LTB4 and cytochrome metabolites of AA/EPA/DHA were also measured. It is possible that different tissues may respond differently to injury but the balance between pro- and anti-inflammatory eicosanoids is somewhat similar. It is noteworthy that PGE2 levels (in conjunction with COXs’ expression) were gradually enhanced from ischemia to reperfusion phase until healing occurs. LXA4 and LTB4 concentrations were elevated during ischemia (up to 2 h) and peaking at 5 h, coinciding with PGE2 levels. These data were taken from ref. [37]. (**A**) Changes in the activities of COX, LOX and CYP enzymes during ischemia and reperfusion injury. (**B**) Changes in the concentrations of LTB4, PGE2, LXA4, 18-HEPE and PGJ2 during ischemia and reperfusion injury/inflammation. Notice the relationship between LTB4 and LXA4 (LTB4 > LXA4 and PGE2 > LXA4) during ischemia and reperfusion injury. It may be noted that LTB4 and PGE2 levels are high that. In turn, trigger the production of LXA4 and 18-HETE and PGJ2 that finally induce resolution of inflammation, tissue regeneration and restoration of homeostasis.

**Figure 10:**
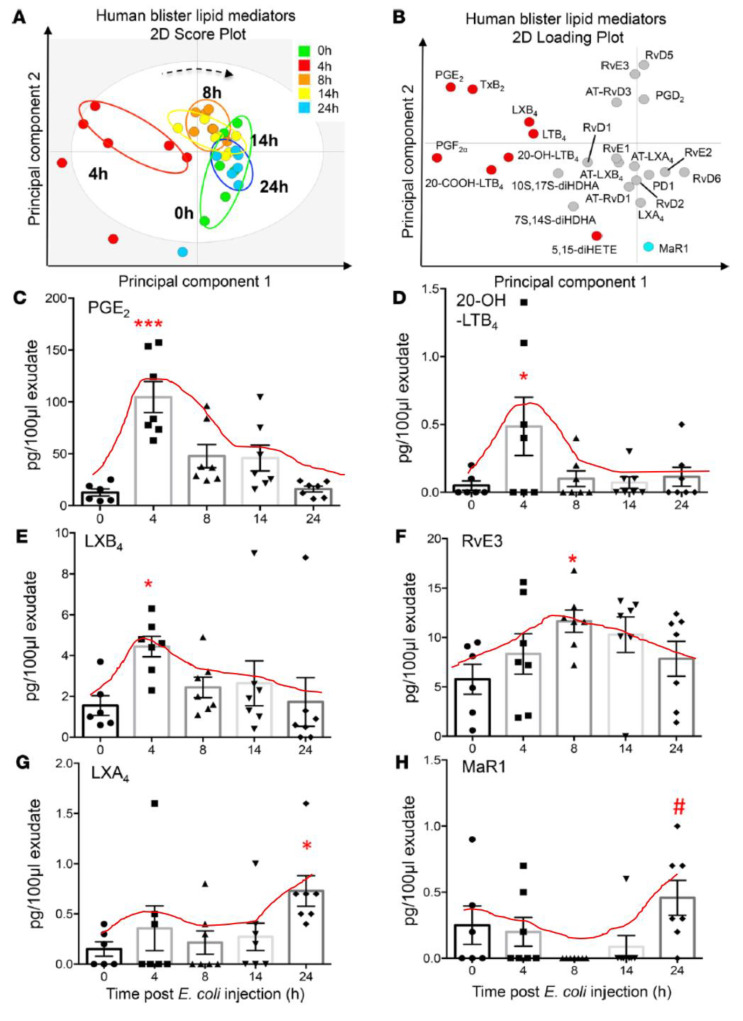
Concentration of both pro- and anti-inflammatory eicosanoids in a human skin model of UV-killed Escherichia coli-driven acute inflammation. It is evident from this study that concentrations of PGE2 and LTB4 coincide with the peaking of LXB4 and LXA4 and the beginning of a gradual but definite increase in those of resolvin E3. Paradoxically, the increase in maresin was not seen until the end of almost 24 h. The results of this study confirm the importance of PGE2 (and of LTB4) as initiators of resolution of inflammation by triggering the initiation of production of anti-inflammatory LXA4 and LXB4, which is supported by the subsequent generation of resolvin E3 (with a gradual increase and decrease from 4 to 24 h), a paradoxical fall in the generation of maresin from 4 to 14 h, and an increase by 24 h. These results also emphasize that there are some specific actions for various eicosanoids in the process of inflammation and its resolution and restoration of homeostasis, though the exact role of each type of eicosanoid needs to be deciphered. These data are from reference [40]. * *p* < 0.05; # *p* < 0.05, *** *p* < 0.05 compared to control.

**Figure 11:**
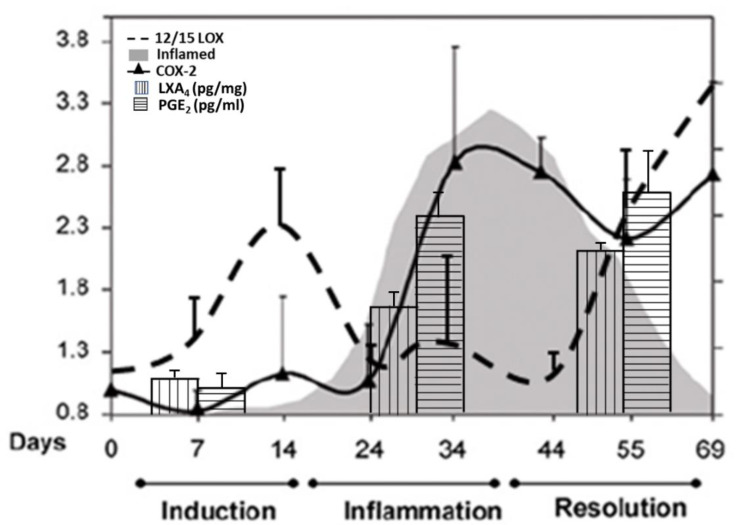
A summary scheme showing potential relationship among COX-2/LOX enzymes and PGE2/LXA4, and induction, active inflammation, and resolution of inflammation based on the data derived from Figure 8, Figure 9 and Figure 10.

GLA, DGLA, AA, EPA, and DHA are potent inhibitors of IL-6 and TNF-α [8,9,10,11,12,26,27,28,29,30,31,32]. PGE2 has both pro- and anti-inflammatory actions. Adequate formation of PGE2 is needed to produce an optimal degree of inflammation to occur that, in turn, activates the ALOX12/15 enzyme by 2- to 2.5-fold to enhance the production of anti-inflammatory LXA4 formation [33,34,35,36]. The activity of the 12/15 ALOX enzyme was boosted during the initiation of the inflammatory phase, returned to a basal level during the inflamed phase, and then surged again on entering the resolution phase of the inflammatory phase that corresponded to the changes in LXA4 levels: no excess LXA4 production during the peak of inflammation but increased as pathogenesis progressed into the resolution phase. Almost 2-fold higher amounts of LXA4 were formed compared to the basal level during the resolution of the inflammation phase. It is surprising that COX-2 inhibition suppressed LXA4 formation, resulting in continuation or perpetuation of inflammation, indicating that COX-2 influences the synthesis of LXA4 (Figure 7) [33,34,35,36]. Similar results have been reported in other inflammatory conditions such as the murine model of autoimmune arthritis [35], intestinal ischemia reperfusion injury [37,38], and in a human skin model of UV-killed *Escherichia coli*-driven acute inflammation [39] (see Figure 8, Figure 9, Figure 10 and Figure 11). The results of these studies suggest that PGE2 (and LTB4) need to be high enough to trigger optimum inflammation that, in turn, triggers the synthesis and release of LXA4 (and possibly resolvins, protectins, and maresins) to initiate and induce the resolution of inflammation [33,34,35,36,37,38,40]. Thus, PGE2 (and LTB4) is critical both to induce inflammation and its resolution. The ability of PGE2 to trigger anti-inflammatory events is further supported by the reports that PGE2, through its receptor EP4, is downregulated in systemic inflammatory conditions such as sepsis [39]. It was found that experimental animals with reduced PGE2 synthesis develop systemic inflammation that is associated with translocation of gut microbiota that could be prevented by EP4 agonists. PGE2-EP4 signals type 3 innate lymphoid cells to produce IL-22 that is needed to inhibit systemic inflammation [39]. These results are supported by the observation that neutrophilic inflammation does not resolve if macrophage-dependent PGE2, which is essential for neutrophil inflammation resolution, is missing. PGE2 seems to signal through the EP4 receptor to augment LXA4 production to induce resolution of inflammation [34]. The ability of PGE2 to enhance LXA4 generation to induce resolution of inflammation is evident from the observation that murine foot pads show increased formation of LXA4 when treated with different concentrations of PGE2 (see Figure 11) [35]. It is noteworthy that even PGE1 enhances LXA4 formation but is much less potent compared to PGE2. These results imply that the anti-inflammatory actions of PGE1 could be due to its ability to enhance LXA4 formation. Similarly, even resolvins D1 (formed from DHA) and E1 (formed from EPA) augment LXA4 formation (see Figure 2, Figure 3 and Figure 4). These results suggest that, at least to some extent, the anti-inflammatory actions of resolvins are brought about by its ability to increase LXA4 formation. As already discussed above, other fatty acids such as GLA, EPA, and DHA have also been shown to increase LX4 formation (see Figure 5), which has been attributed to their (GLA, EPA and DHA) ability to displace AA from the cell membrane phospholipid pool that, in turn, is converted to LXA4. These results suggest that several lipids (such as GLA, EPA, DHA, and resolvins D1, E1, PGE1, and PGE2) are all capable of enhancing LXA4 formation that may underlie the anti-inflammatory action of these compounds. Hence, it is opined that LXA4 is the common mediator of the anti-inflammatory actions of GLA, AA, EPA, DHA, PGE1, PGE2, and resolvins (and, possibly, protectins and maresins). In other words, the ability of a compound to enhance LXA4 formation may determine its degree of anti-inflammatory action: the higher the capacity of a compound to trigger the formation of LXA4, the higher its ability to produce anti-inflammatory action (LXA4 > resolvins ≥ protectins ≥ maresins > DHA > EPA > GLA ≥ PGE1). 

The importance of PGE2 is further illustrated from the observation that it is needed for tissue regeneration. For instance, inhibition of 15-PGDH (15-prostaglandin dehydrogenase, a prostaglandin-degrading enzyme) not only produced a 2-fold increase in PGE2 concentrations in the bone marrow, colon, and liver, but also enhanced hematopoietic capacity. The 15-PGDH-deficient animals showed a rapid liver regeneration after partial hepatectomy and enhanced recovery of neutrophils, platelets, and erythrocytes [41]. This is supported by other studies, which showed that PGE2 is needed to promote hematopoiesis and restore hemostasis [42,43,44,45,46]. These results imply that, perhaps, administration of AA, the precursor of both PGE2 and LXA4, may lead to the formation of LXA4 to induce inflammation resolution and PGE2 to trigger tissue regeneration at an appropriate time to augment tissue regeneration. 

Since both PGE2 and LXA4 are formed from AA, it is likely that AA metabolism is crucial to the inflammatory process and its resolution. Thus, the balance between PGE2 and LXA4 (a similar balance may exist between PGE3/LTE5 and resolvins, protectins, and maresins but has not been studied so far) and the tissue concentrations of GLA, DGLA, AA, EPA, and DHA (as modulators of IL-6 and TNF-α production and action) is crucial to determine the final status of the inflammatory events. GLA, DGLA, AA, EPA, and DHA are not only important constituents of the cell membrane but also modulate its fluidity that, in turn, influences the expression of several receptors and their binding properties. PGE2 and LTs facilitate generation of M1 macrophages (which are pro-inflammatory in nature), whereas GLA, DGLA, AA, EPA, DHA, PGE1, LXA4, resolvins, protectins, and maresins favor generation of M2 macrophages [47,48,49,50,51,52,53,54] (which are anti-inflammatory in nature), which may be relevant to the involvement of these bioactive lipids in COVID-19. It is interesting to note that the M1 phenotype is associated with 6-keto PGF1α, PGE2, and LTB4 [54]. Since PGE2 is associated with both M1 and M2 macrophages, it can be argued that, in the early phase of inflammation, when PGE2 concentrations are still peaking, it (PGE2) facilitates M1 generation. In contrast, when PGE2 levels have reached their peak it triggers the inflammation resolution process by stimulating LXA4 formation, and it (PGE2) facilitates M2 generation [48,49,50,51,52,54]. 

As discussed above, the induction of an adequate degree of inflammation is needed to trigger inflammation resolution events and restoration of homeostasis [33,34,35,36,37,38,39,40]. PGE3 and LTs of 5 series formed from EPA are less pro-inflammatory compared to PGE2 and LTs of 4 series formed from AA [10]. This implies that PGE3 and LTs of 5 series do not trigger inflammation of a sufficient degree to initiate the inflammation resolution process. Hence, it is likely that resolvins, protectins, and maresins derived from EPA and DHA may be inadequate to trigger an efficient inflammation resolution process even though they are potent anti-inflammatory compounds. Previously, we showed that EPA and DHA displace AA from the cell membrane lipid pool to enhance the formation of LXA4 (see Figure 5). But LXA4 formed as a result will be insufficient to induce full remission of the inflammatory process. This is so since the concentrations of LXA4 formed are much lower compared to those formed following AA supplementation [13]. It is noteworthy that LXA4 generation is enhanced by resolvin treatment [14,16] (see Figure 2 and Figure 3, and Figure 4, Figure 5 and Figure 6 for anti-inflammatory actions of AA and LXA4). Based on these results, it is proposed that the formation of adequate amounts of LXA4 is critical for the resolution of inflammation. 

**Figure 12:**
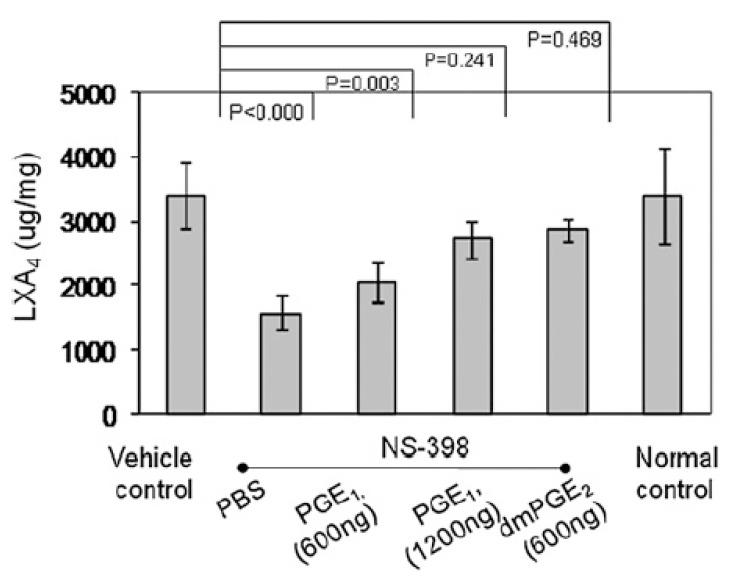
Effect of PGE1 and PGE2 on the generation of LXA4. This study was performed in the foot pads of the murine collagen-induced arthritis. It is evident form this data that PGE1 and PGE2 can enhance LXA4 formation. PGE2 is more potent than PGE1 to induce LXA4 generation when tested at the same concentration. These data are taken from reference [35].

**Figure 13:**
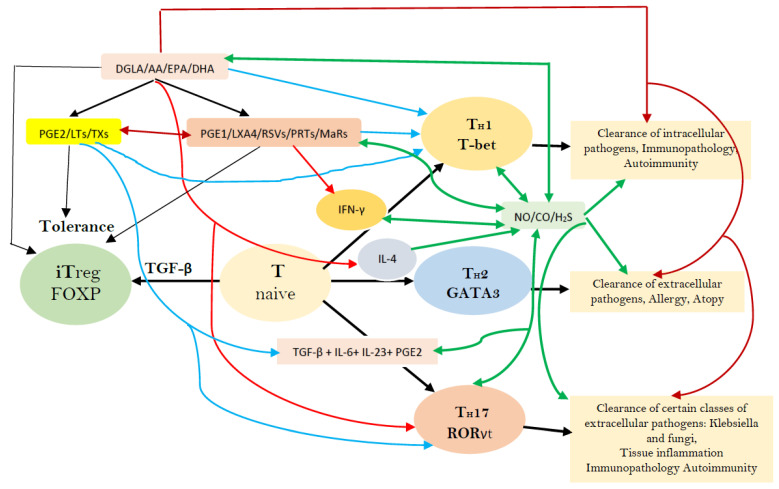
Factors that have a regulatory role in the formation of different subsets of T helper cells. RSVs = Resolvins; PRTs = Protectins; MaRs = Maresins; LXA4 = Lipoxin A4; LTs = Leukotrienes; TXs = Thromboxanes; NO = Nitric oxide; CO = Carbon monoxide; H_2_S = Hydrogen peroxide; TGF-β = Transforming growth factor β; IFN-γ = Interferon-γ. Naive CD41 T cells differentiate into T helper cells: T_H_1, T_H_2, and T_H_17. TGF-β converts naive T cells into FOXP3-expressing induced Treg (iTreg) cells. T helper cell differentiation needs T-bet, GATA3, and ROR-γt. Terminally differentiated T helper cells produce a specific combination of effector cytokines needed for the adaptive immune system. TGF-β, retinoic acid, or cytokines: IL-6, IL-1, IL-23, or IL-27 secreted by the innate immune system cells (immature or activated dendritic cells (DCs), respectively) dictate whether a naive T cell develops into a FOXP31 Treg cell, a T_H_17 cell, or otherwise. PGE2 through its receptor EP4 on T cells and dendritic cells facilitates T_H_1 cell differentiation and amplifies IL-23-mediated T_H_17 cell expansion. Bioactive lipids modulate the generation, proliferation, and function of several immunocytes, and their secretion of soluble mediators and nitric oxide (NO)/carbon monoxide (CO)/hydrogen sulfide (H_2_S) has a modulatory action on various immunocytes and their actions, as shown in the figure. This figure is taken from reference [56]; for more details, see reference [56].

Both pre-treatment (exposing the RIN5F cells initially to resolvin/protectin/LXA4 and then to alloxan) and simultaneous treatment (RIN5F cells were exposed to LXA4/resolvin/protectin and alloxan at the same time) studies showed that LXA4 is the more potent compared to resolvins and protectins in preventing alloxan-induced cytotoxicity to RIN5F (rat insulinoma) cells in vitro [17]. Similarly, LXA4 was more potent compared to resolvins and protectins in preventing the cytotoxic action of benzo(a)pyrene, streptozotocin, and doxorubicin [13,14,15,17]. In addition, AA showed potent anti-inflammatory action by virtue of its ability to suppress IL-6 and TNF-α production and the expression of NF-κB and prevented both alloxan- and streptozotocin-induced type 1 and type 2 diabetes mellitus in experimental animals [13,17,55]. This anti-inflammatory action of AA seems to be brought about, at least, in part, by enhancing the formation of LXA4. Thus, PGE2 and LXA4 derived from AA appear to be the most suited to induce inflammation and initiate and resolve inflammation in an optimal fashion (see Figure 7, Figure 8, Figure 9, Figure 10, Figure 11, Figure 12 and Figure 13).

## 6. PGE2 and LXA4 Regulate Inflammation and Its Resolution 

It is believed that AA is harmful, with the impression that its administration enhances PGE2 formation, a pro-inflammatory molecule. It is noteworthy that LXA4, a potent anti-inflammatory compound, is also derived from AA [10,16,56,57,58,59]. AA can be given orally (840 to 2000 mg/day) for 50 days to healthy individuals with no increases in inflammation or related metabolic activities [60]. AA can be administered intravenously when prepared suitably [61]. AA supplementation to animals and humans enhanced its tissue content with no change in PGE2 levels, but an increase in LXA4 formation was reported [62,63,64]. My own studies and those of others [13,17,33,65,66,67,68] revealed that AA has potent anti-inflammatory action. Like AA, LXA4 prevented the development of alloxan- and streptozotocin-induced type 1 and type 2 diabetes mellitus, respectively, and suppressed IL-6 and TNF-α production and NF-kB expression. 

Paradoxically, PGE2 seems to have an anti-inflammatory action as well. PGE2 suppresses IL-6 and TNF-α production and alters macrophage polarization induced by mesenchymal stem cells (MSCs) [69,70,71,72,73,74,75]. PGE2 binds to both EP2 and EP4 receptors depending on its concentration. At low concentrations, PGE2 binds to EP4, a high affinity receptor, and enhances the production of IL-23, whereas high PGE2 amounts bind to EP2 receptor to inhibit IL-23 production [76]. Furthermore, PGE2 triggers the production of LXA4 and inhibits LTB4 synthesis by modulating the expression of 5- and 15-lipoxygenases and, thus, triggers the anti-inflammatory pathway to induce resolution of inflammation [35,36]. This interaction and crosstalk among macrophages, neutrophils, PGE2, LXA4, and LTB4 are essential to trigger the much needed inflammation and initiate inflammation resolution. This redirection of the AA metabolism from PGE2/LTB4 to LXA4 seems to depend on the biphasic release of AA from the cell membrane lipid pool. The first phase of AA release in response to iPLA2 (calcium-independent phospholipase A2) activation results in the conversion of AA to PGE2, whereas the second wave of AA release in response to cPLA2 and sPLA2 (cytosolic and soluble phospholipase A2, respectively) activation results in the generation of LXA4. This specific and unique process of utilization of released AA to form either PGE2/LTB4 or LXA4 regulates inflammation and its resolution that is partly dependent and regulated by the local concentrations of IL-1β, IL-4, and IL-10 [33]. 

## 7. Critical Role of AA in Inflammation Resolution 

**Figure 14:**
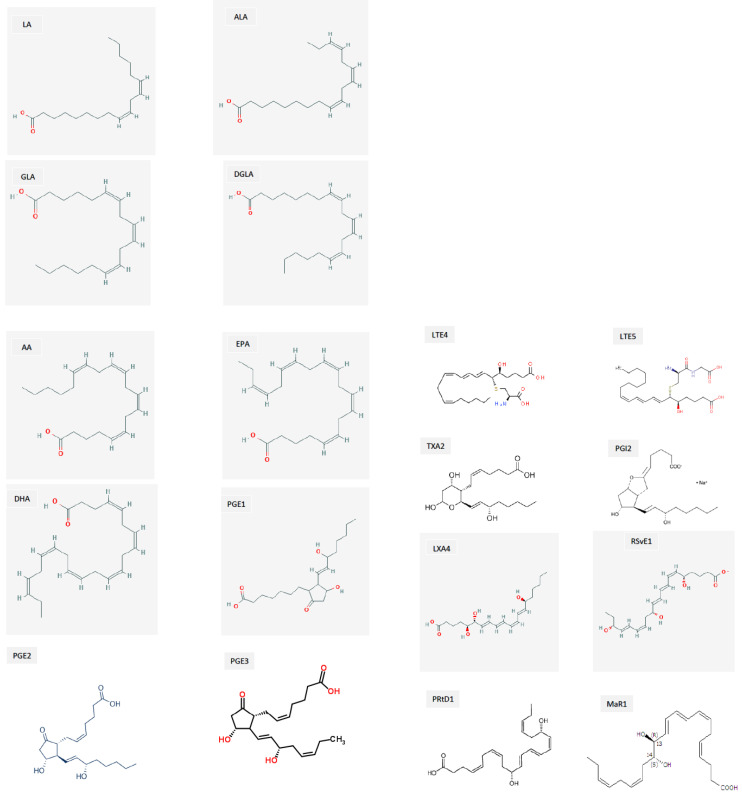
Structures of various BALs. Abbreviations: LA = Linoleic acid; ALA = Alpha-linolenic acid; GLA = Gamma-linolenic acid; DGLA = Dihomo-gamma-linolenic acid; AA = Arachidonic acid; EPA = Eicosapentaenoic acid; DHA = Docosahexaenoic acid; PGE1, PGE2, and PGE3 = Prostaglandin E1, E2, and E3, respectively; TXA2 = Thromboxane A2; LTE4 and LTE5 = Leukotriene E4 and E5, respectively; PGI2 = Prostacyclin; LXA4 = Lipoxin A4; RSvE1 = Resolvin E1; PRtD1 = Protectin D1; Mar1 = Maresin1.

Under normal physiological conditions a delicate balance is maintained between pro-inflammatory T_H_1 (IL-2, IFN-γ) and anti-inflammatory T_H_2 cytokines (IL-4, IL-5, IL-10, IL-13) to regulate inflammation. IFN-γ-producing CD4+ T_H_1 cells and PGE2 are needed to control invading microbial inflammatory stimuli to initiate and maintain the mononuclear inflammatory response. IL-17 (T_H_17), a pro-inflammatory cytokine, formation is dependent upon IL-23 and PGE2, which induces chemokine expression and recruitment of cells (see Figure 14) [33,77,78,79,80,81,82]. PGE1, LXA4, resolvins, protectins, and maresins, by virtue of their negative feed-back control on the production of PGE2, LTs, and thromboxanes (that have pro-inflammatory actions), inhibit the formation of T_H_17 cells and their production of pro-inflammatory IL-17, IL-22, and IL-23 in view of the close interaction among these cytokines (IL-17, IL-22, IL-23) [82,83,84,85,86]. Once the purpose of inflammatory response is achieved, IL-10, IL-4, and LXA4 (and, possibly, resolvins, protectins, and maresins) are produced to initiate and induce the resolution of inflammation [33,34,35,36,37,38,39,40]. Thus, PGE2 (LTs and thromboxanes) and LXA4 (and the other anti-inflammatory bioactive lipids, PGE1, resolvins, protectins, and maresins) are needed to initiate and resolve inflammation, respectively. 

AA and LXA4 suppress NF-κB expression that is essential for enhancing the IL-17 and IL-23 production [10,13,16,17,33,40,87,88,89] that seems to play a major role in COVID-19 [90,91,92,93,94]. IL-6, TNF-α, and IL-17 enhance iNOS (inducible nitric oxide) expression that, in turn, augments PD-L1 (programmed death-ligand 1) expression in MSCs (mesenchymal stem cells) [95,96,97]. This renders MSCs to be recognized as self that is essential to facilitate their (MScs’) survival, proliferation, and differentiation to restore homeostasis after the inflammation has resolved and all the debris at the site of injury has been removed [98]. However, this action of IL-17 on MSCs and their expression of PDL-1 may also have an unwelcome consequence, namely, failure/suppression of recognition of SARS-CoV-2 and other microbial-infected cells as non-self and mount an immune attack to eliminate them. As a result, the infected cells may survive and aid in the proliferation and spread of infection to other cells/tissues. 

Resolvin E1 and E2, derived from EPA, resolvins of D series, and protectins and maresins derived from DHA have anti-inflammatory actions like LXA4 and suppress IL-23 and IL-17 production in addition to their ability to inhibit IL-6 and TNF-α [99,100,101,102]. Resolvin E1 promotes LXA4 production, implying that LXA4 is the mediator of the actions of resolvin E1 [14,15,16] (see Figure 2, Figure 3 and Figure 4). LXA4 attenuates liver fibrosis by suppressing TNF-α, IFN-γ, IL-2, and IL-17 and augmenting IL-4 and IL-10 production [103], implying that LXA4 and its precursor AA may be of benefit in preventing the lung fibrosis seen in some patients with COVID-19 (termed as long-haulers). This is in addition to the ability of LXA4/AA to suppress acute lung injury [104,105]. In this context, it is noteworthy that the beneficial actions of MSCs and other types of stem cells in the management of SARS-CoV-2 infection (COVID-19) and other inflammatory conditions could be attributed to their ability to secrete PGE2 and LXA4 as needed [69,70,71,72,74,75,106,107,108,109,110,111,112,113,114,115,116,117]. 

My studies revealed that the inhibitory action of AA and LXA4 on the production of IL-6 and TNF-α is to the same extent that was exerted by resolvin E1, implying that LXA4 is as potent as resolvin E1 (and resolvin D1, unpublished data) in its anti-inflammatory action (see Figure 2, Figure 3, Figure 4, Figure 5 and Figure 6) [13,14,15,17]. AA, the precursor of LXA4 and PGE2, prevented streptozotocin (STZ)-induced type 1 and type 2 diabetes mellitus and suppressed plasma IL-6 and TNF-α levels and the pro-inflammatory gene NF-κB to the same extent as that of LXA4 (see Figure 6 and Figure 7). AA treatment also restored plasma LXA4 levels to normal by augmenting the expression of COX-2 and 12-LOX genes (see Figure 6 and Figure 7). Since resolvins enhance the production of LXA4 to bring about their anti-inflammatory and cytoprotective actions [14,15,16] (see Figure 2, Figure 3 and Figure 4), it is reasonable to suggest that a crosstalk occurs between n-3 and n-6 fatty acids’ metabolism in such a way that LXA4 production is enhanced to resolve inflammation. This implies that plasma and tissue concentrations of AA are critical to achieve optimal inflammation resolution. 

EFA metabolism is modulated by several factors including but not limited to folic acid; vitamins B1, B2, B6, and B12; vitamin C; cholesterol; dietary saturated fats; trans fats; ethanol; iron; magnesium; zinc: and drugs such as statins (see Figure 1). Folic acid, B1, B2, B6, B12, and magnesium are co-factors needed for optimal activity of desaturases, whereas vitamin C is needed for the formation of PGE1 from DGLA. Insulin activates desaturases. Saturated (including cholesterol) and trans fats inhibit the activity of desaturases. A deficiency of GLA, DGLA, AA, EPA, and DHA due to low activity of desaturases increases the rigidity of cell membrane, as seen in those with diabetes mellitus, hypertension, and coronary heart disease, who have decreased plasma and tissue concentrations of GLA, DGLA, AA, EPA, and DHA [8,9,10,11,12,18,19,20,21,22]. Hyperglycemia inhibits the activities of desaturases, leading to a decrease in the formation of GLA, DGLA, AA, EPA, and DHA even if the dietary intake of LA and ALA are adequate. This may result in a decreased formation of PGE1 (from DGLA), PGI2, PGJ2, and LXA4 (from AA), resolvins (from EPA and DHA), and protectins and maresins (from DHA) that are needed to suppress inappropriate inflammation.

## 8. AA and Other Bioactive Lipids Possess Antimicrobial Action

Whenever human cells and tissues are invaded by microbes, the host immune system is activated to eliminate or neutralize them by generating specific antibodies, immunocytes elaborate suitable cytokines and reactive oxygen species (ROS), activate complement system, and other specific and non-specific means to trap and kill them. In this process there could occur little or no injury to various tissues, though some amount of inflammation, injury, and cell/tissue damage is inevitable. The elimination of invading microbes and the resolution of inflammation and subsequent repair of the tissue damage must occur in a coordinated and orderly fashion. This can occur only if there is a crosstalk among the invading organisms, the host tissues under attack, and the immune system, as discussed above. In addition, when the microbes invade the cells/tissues, they invariably perturb/disrupt membrane integrity during cell entry, leading to the activation of phospholipase A2 (PLA2) that induces the release of various bioactive lipids (BALs) such as LA, GLA, DGLA, AA, ALA, EPA, and DHA. These lipids have anti-microbial actions and can act intracellularly through one of five membrane G protein-coupled receptors (GPCRs) in immune cells, resulting in diverse effects on innate immune function. Studies revealed that BALs can inactivate or kill *Staphylococcus aureus* and coagulase-negative staphylococci, group A streptococci, fungi, and enveloped viruses, including influenza and HIV [118,119,120,121,122,123,124,125,126,127,128,129,130,131,132,133,134,135,136]. Of all the fatty acids tested, AA seems to be the most potent and crucial in inducing not only the anti-microbial action of macrophages, NK cells, and other immunocytes but also the pathobiology of inflammation and its resolution, as discussed above. AA and other fatty acids seem to induce their antimicrobial action by their ability to induce leakage and even lysis of microbial cell membranes (including disruption of viral protein envelopes), as well as various cellular metabolic effects, including but not limited to inhibition of respiratory activity, effects on transportation of amino acids, and uncoupling of oxidative phosphorylation [133,134,135,136,137]. In addition, *Staphylococci* in the lung alveoli are killed mainly outside alveolar macrophages that resides in the AA present in the surfactant [119,120,121,122,123]. 

It has been proposed that alveolar macrophages, leukocytes, T and B cells, NK cells, and other immunocytes release AA and other unsaturated fatty acids into their surrounding milieu when exposed to various microbes including the viruses SARS-CoV-2, SARS, and MERS to inactivate them and, thus, protect the lungs and other tissues. This fundamental mechanism seems to be employed by the human body to prevent and protect itself from various invading microbes. Thus, it is reasonable to suggest that a deficiency of AA and other unsaturated fatty acids may render a person susceptible to various infections by viruses such as SARS-CoV-2, SARS, and MERS [138,139,140,141].

## 9. BALs Prevent Microbial Infection Including SARS-CoV-2

**Figure 15:**
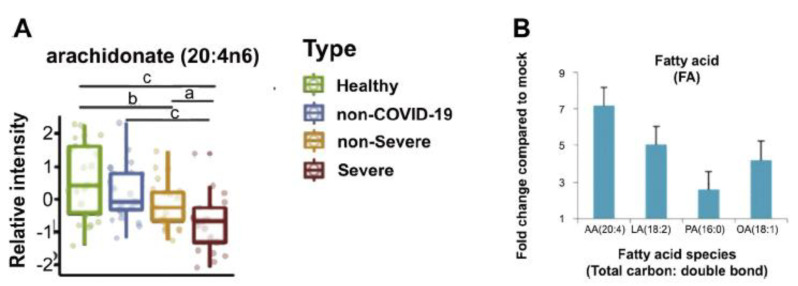
(**A**) Plasma levels of AA in those infected with SARS-CoV-2. (**B**) Fold change compared to mock. These results show that those infected with the virus have low levels of AA. These data are taken from Reference [144]. ^a, b, c^ *p* < 0.05 compared to respective controls, as shown in the figure.

**Figure 16:**
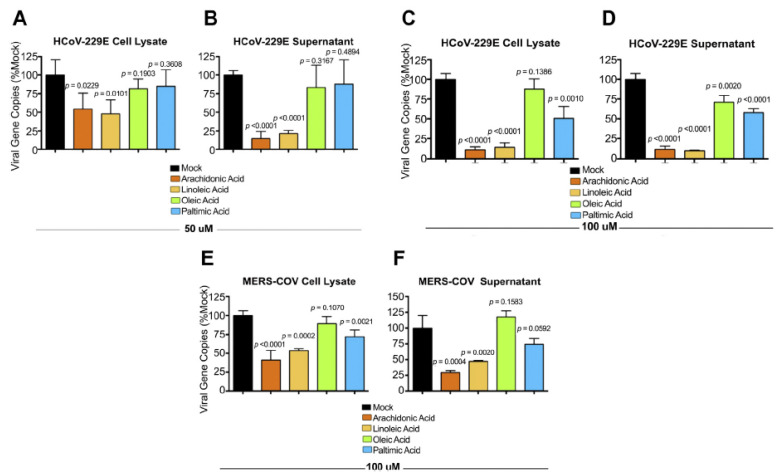
Ability of LA and AA and other fatty acids to inactivate HCoV-229E and MERS-CoV viruses. HuH-7 cells were infected with HCoV-229E or MERS-CoV viruses. After 1 hour of inoculation with the virus, the cells were treated with 50 or 100 mmol of fatty acids for 24 h. Both supernatant and cell lysates were collected and analyzed by RT-qPCR technique. *p* < 0.05. These data are taken from reference [145]. (**A**,**B**) shows the number of viral gene copies after treatment with 50 uM of various fatty acids of Huh-7 cells infected with HCoV-229E virus in their cell lysate and cell culture supernatant respectively. (**C**,**D**) Same study but performed using 100 uM of fatty acids. (**E**,**F**) Similar study performed with MERS-CoV virus.

In addition to their participation in inflammation, resolution of inflammation, and ability to modulate immune response, as discussed above, it is interesting to know that cryo-electron microscopy studies showed that the SARS-CoV-2 spike protein receptor binding domain binds tightly to LA (linoleic acid, 18:2 n-6), an essential fatty acid, rendering it locked in S conformation that reduces the interaction and binding of the spike protein to ACE2 [142]. This is expected to reduce the infectivity of the SARS-CoV-2. A similar reduced infectivity of MERS-CoV was also noted in relation to LA. This was further supported by the report that ALA, EPA, and DHA (and, possibly, AA in view of their structural similarity, see Figure 14 for the structures of these fatty acids) effectively interfere with the binding of SARS-CoV-2 with human ACE2, the receptor for SARS-CoV-2 [143]. In addition, ALA and EPA not only significantly blocked the entry of SARS-CoV-2 to a spike protein pseudo-virus, but also reduced the activity of TMPRSS2 and cathepsin L-proteases that are needed for the infectivity of SARS-CoV-2. In this study [143], it was noted that LA, ALA, EPA, and DHA could reduce the binding of SARS-CoV-2 to the human ACE2 receptor to ~4–5% of the control. It is noteworthy that, in cell culture studies, LA synergized with remdesivir in markedly suppressing SARS-CoV-2 replication [142]. Furthermore, it was reported that, when human cells are exposed to SARS-CoV-2 and/or human corona virus 229E (HCoV-229E), the human cells release significant amounts of LA and AA to inactivate the viruses [144,145] (See Figure 15 and Figure 16). It was shown that supplementation of LA and AA to HCoV-229E- and MERS-CoV-infected Huh-7 cells significantly suppressed virus replication. These results imply that BALs can inactivate and decrease the infectivity of SARS-CoV-2 and related viruses. Based on these results, it is suggested that, when human cells are exposed to SARS-CoV-2 virus, activation of PLA2 can occur, which leads to release of LA and AA from the cell membrane phospholipid fraction to specifically inactivate the SARS-CoV-2 and related viruses. In the event this release of LA and AA and other BALs is inadequate, it could result in replication of SARS-CoV2 and result in COVID-2. These results are in support of the proposal that BALs play a significant role in the pathobiology of COVID-2 [138,139,140,141]. 

## 10. Conclusions and Therapeutic Implications 

Based on the preceding discussion, it is evident that BALs play a significant role in the pathobiology of COVID-19 and similar diseases. BALs (especially DGLA, AA, EPA, and DHA) and their metabolites regulate inflammation and the associated immune response against various invading microbes including SARS-CoV-2 and in the resolution of the inflammation induced by these infections in a timely and regulated fashion. It is important that inflammation needs to occur to an optimum degree so that its resolution is triggered in an orderly fashion. This is clear from the observation that PGE2, a pro-inflammatory molecule that triggers inflammation to start with, can initiate the formation of much needed LXA4 to induce the resolution of inflammation and restore homeostasis. LXA4 not only inhibits PGE2 formation but also suppresses LTB4 (a pro-inflammatory molecule derived from AA and LTB5, derived from EPA) synthesis by enhancing the expression of 5- and 15-lipoxygenases [33,34,35,39]. Furthermore, resolvins that are needed to induce resolution of inflammation enhance the synthesis of LXA4 [33,35]. 

**Figure 17:**
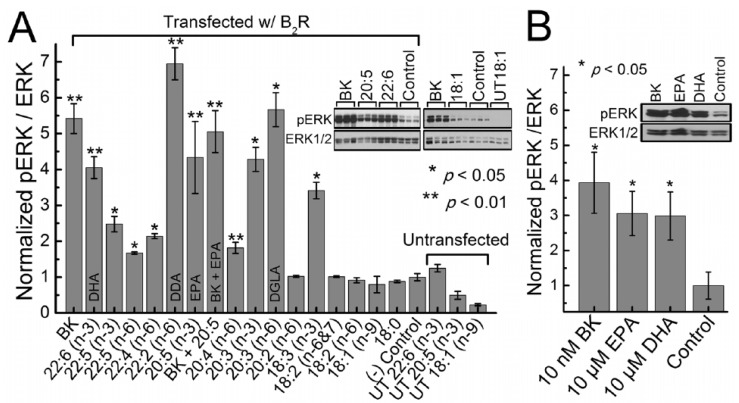
Activation of B2R by various fatty acids in HEK293 cells. Bradykinin concentration in (**A**) was 100 nM (positive control) and (**B**) was 10 nM. Fatty acid concentrations were 10 μM in HEK 293 cells transfected with B2R. It is evident from these studies that, while EPA, DHA, and DGLA significantly enhanced pERK/ERK, AA failed to do so, implying that AA suppresses bradykinin receptor activation, especially in comparison to EPA and DHA. These data are taken from reference [148]. * *p* < 0.05; ** *p* < 0.01.

PGE2 is not only needed for optimal inflammation but is essential for tissue regeneration [41,42,43,44,45] and to restore homeostasis. This implies that timely administration of AA, the precursor of PGE2 and LXA4, not only suppresses excess production of IL-6 and TNF-α to prevent the ‘cytokine storm’ seen in moderate to severe cases of COVID-19 but also restores recovery of hematopoiesis and immune response due to suppression of geminal centers seen in them [4,146,147]. At this juncture, it is important to note that Garvin et al. [148] proposed that excess bradykinin production causes an increase in vascular dilation, vascular permeability, and hypotension that could be responsible for critical illness in those with COVID-19. Candelario et al. [149] showed that PUFAs increase membrane fluidity and alter B2R (bradykinin 2 receptor) activation (see Figure 17). Of all the unsaturated fatty acids tested (especially DGLA, EPA, DHA, and AA), AA was found to suppress bradykinin receptor activation, whereas EPA, DHA, and DGLA activated the same. Based on these results, it is reasonable to propose that AA suppresses bradykinin receptor activation and, thus, inhibits the ‘bradykinin’ storm. These results, coupled with the observation that BALs (especially LA, ALA, AA, EPA, and DHA) can suppress or inactivate SARS-CoV-2 and similar viruses [142,143,144,145], lend further support to the concept that bioactive lipids, especially AA, not only participate in the pathobiology of COVID-19 but also suppress both “cytokine and bradykinin storms”.

Mann et al. [150] presented evidence that those with severe COVID-19 have a shift in the neutrophil-to-T cell ratio, elevated serum IL-6, MCP-1, and IP-10, and modulation of CD14+ monocyte phenotype and function including poor induction of the COX-2 enzyme, implying suboptimal production of PGE2. Longitudinal analysis showed that some of these features revert to normal in those who recovered from the disease. Patients with mild COVID-19 showed higher TNF-α and COX-2 expression in LPS-activated monocytes compared to patients with severe disease. COX-2 expression remained low in severe COVID-19 patients throughout the intensive care period, but levels reverted to normal upon recovery. These results suggest that supplementation of AA may lead to an increase in COX-2 expression and generation of higher amounts of PGE2 that may benefit these patients. 

**Figure 18:**
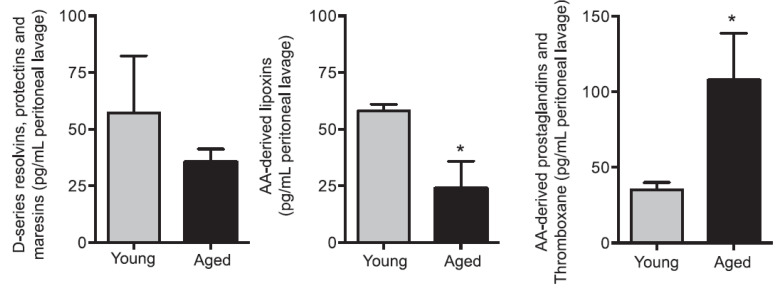
Aged mice show reduced resolvins, protectins, and maresins and LXA4 in the peritoneal lavage of zymosan-challenged animals. * *p* < 0.05 compared to young mice. These data are taken from reference [151].

Aging decreases LXA4 levels (LXA4 > resolvins, protectins, and maresins) with a relatively high content of PGs, and thromboxanes that have pro-inflammatory action (see Figure 18) was reported [151], suggesting a decrease in AA content. In such an instance, supplementation of AA with or without EPA/DHA may be necessary to enhance LXA4 formation and suppress inappropriate PGE2 (and other PGs and TXA2 and LTE4) production. Since the decrease in LXA4 formation is more dominant compared to resolvins, protectins, and maresins, perhaps supplementation of only EPA/DHA may be not advisable. 

**Figure 19:**
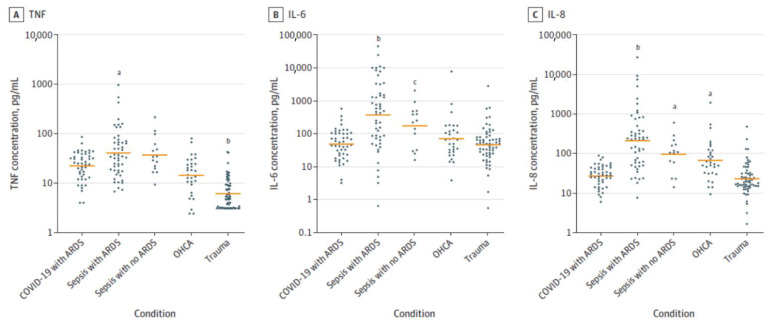
Cytokine levels in critically ill patients with COVID-19 and other conditions. Plasma concentrations of TNF (**A**), IL-6 (**B**), and IL-8 (**C**) in patients with COVID-19 and ARDS (*n* = 46), septic shock with ARDS (*n* = 51), septic shock without ARDS (*n* = 15), out-of-hospital cardiac arrest (OHCA) (*n* = 30), and multiple traumas (*n* = 62). ^a^ *p* < 0.01 vs. COVID-19 with ARDS; ^b^ *p* < 0.001 vs. COVID-19 with ARDS; ^c^ *p* < 0.05 vs. COVID-19 with ARDS. These data are taken from reference [5].

Furthermore, the very occurrence of a ‘cytokine storm’ in severe COVID-19 is being questioned. It was reported that those who are critically ill due to COVID-19 have much lower levels of cytokines compared to those who have sepsis [5] (see Figure 19). This emphasizes the importance of restoring the concentrations of various lipids including PGE2 and LXA4 to physiological levels that will restore inflammation and immune response abnormalities to normal in COVID-19. These results imply that AA administration may be of significant benefit in other severely ill patients, such as those with sepsis. 

It was reported that patients with COVID-19 have a severe deficiency of vitamin C [152]. This suggests that co-administration of vitamin C to those with COVID-19 is important. Vitamin C enhances the formation of PGE1, an anti-inflammatory, anti-platelet anti-aggregator, and modulates immune response [153,154,155], actions that are remarkably like those shown by LXA4. Insulin has anti-inflammatory actions [156,157,158]. These results suggest that administration of adequate amounts of AA/EPA/DHA (in the right proportion), vitamin C, and insulin to those patients with severe COVID-19 may be important even if they are not having significant hyperglycemia. 

Subjects with co-morbid conditions such as hypertension, diabetes mellitus, and coronary heart disease are known to have high morbidity and mortality due to COVID-19. Previously, I showed that these patients have low plasma concentrations of AA, EPA, and DHA and, so, are likely to suffer from a deficiency of LXA4, resolvins, protectins, and maresins [9,11,13,14,15,16,17,18,19,20,21,22,23,24,55,58,159]. Once again, this evidence suggests that AA/EPA/DHA deficiency and reduced formation of their anti-inflammatory molecules render them more susceptible to develop COVID-19 and its associated complications. 

The proposal made here and elsewhere [138,139,140,141] can be verified by studying whether GLA, DGLA, AA, EPA, DHA, LXA4, resolvins, protectin, and maresins can inactivate SARS-CoV-2. It is necessary to evaluate the role of desaturases, COX-2, -5, and -12, and 15-LOX enzymes and different types of phospholipases in COVID-19. In patients with COVID-19, plasma levels of various EFAs and their metabolites can be measured to know whether their deficiency occurs in COVID-19. Potential therapeutic benefits of EFAs and their metabolites need to be studied in well-designed clinical studies. It is necessary to know whether EFAs and their metabolites influence the development of antibodies against SARS-CoV-2. Since EFAs and their metabolites have a broad spectrum of actions, it is important to design studies with care and arrive at the correct combination of AA/EPA/DHA that can prevent and ameliorate COVID-19. 

## Figures and Tables

**Table 1:** Content of fatty acids in normal liver and hepatoma cells and in microsomal suspensions from normal liver and Yoshida hepatoma cells. All values of mean ± S. E.

Measurement (Fatty Acid)	Normal Intact Liver	Intact Yoshida Cells	Normal Liver Microsomes	Yoshida Microsomes
20:4 (AA)	16.7 ± 2.4	8.7 ± 0.7	19.1 ± 2.4	9.6 ± 0.8
22:6 (DHA)	6.3 ± 0.2	5.2 ± 0.6	6.1 ± 0.3	5.3 ± 0.4

**Table 2:** The percentage of distribution of fatty acids from plasma phospholipid fraction in patients with hypertension (HTN), coronary heart disease (CHD), type 2 diabetes mellitus, and diabetic nephropathy that are common with advanced age.

Fatty Acid	Control	HTN	CHD	Type 2 DM	Diabetic Nephropathy
18:2 n-6 (LA)	18.6 ± 3.1	14.5 ± 3.1 *	17.8 ± 5.0	13.9 ± 5.3	15.1 ± 3.1
18:3 n-6 (GLA)	0.14 ± 0.1	0.4 ± 0.3 *	0.1 ± 0.1 *	0.2 ± 0.3	0.1 ± 0.2
20:3 n-6 (DGLA)	3.4 ± 1.0	3.1 ± 0.9	2.7 ± 1.1	1.7 ± 1.0 *	2.0 ± 0.8 *
20:4 n-6 (AA)	9.4 ± 1.8	7.8 ± 2.0 *	7.0 ± 2.1 *	4.6 ± 1.8 *	6.6 ± 2.6 *
22:5 n-6	0.7 ± 0.4	0.4 ± 0.4 *	1.0 ± 0.9	2.1 ± 0.6 *	1.3 ± 0.5 *
20:4 n-6/18:2 n-6	0.51	0.54	0.39	0.33	0.43
18:3 n-3 (ALA)	0.2 ± 0.1	0.4 ± 0.2 *	0.3 ± 0.5	0.1 ± 0.2 *	0.1 ± 0.1 *
20:5 n-3 (EPA)	0.4 ± 0.4	0.6 ± 0.6	0.1 ± 0.2 *	0.3 ± 0.3	0.2 ± 0.3
22:5 n-3	0.5 ± 0.2	0.4 ± 0.5	0.3 ± 0.3 *	1.6 ± 1.3	1.7 ± 1.1
22:6 n-3 (DHA)	1.4 ± 0.5	1.2 ± 0.6	0.8 ± 0.4 *	0.5 ± 0.4 *	0.5 ± 0.3 *
20:5 n-3/18:3 n-3	1.8	1.39	0.41	3.2	4.0

All values are expressed as mean ± S. D. * *p* < 0.05 compared to control. These data are taken from ref. [9].
